# Endoplasmic Reticulum Stress and Insulin Biosynthesis: A Review

**DOI:** 10.1155/2012/509437

**Published:** 2012-03-05

**Authors:** Mi-Kyung Kim, Hye-Soon Kim, In-Kyu Lee, Keun-Gyu Park

**Affiliations:** ^1^Department of Internal Medicine, Keimyung University School of Medicine, Daegu 700-712, Republic of Korea; ^2^World Class University Program, Department of Internal Medicine, Kyungpook National University School of Medicine, Daegu 700-721, Republic of Korea

## Abstract

Insulin resistance and pancreatic beta cell dysfunction are major contributors to the pathogenesis of diabetes. Various conditions play a role in the pathogenesis of pancreatic beta cell dysfunction and are correlated with endoplasmic reticulum (ER) stress. Pancreatic beta cells are susceptible to ER stress. Many studies have shown that increased ER stress induces pancreatic beta cell dysfunction and diabetes mellitus using genetic models of ER stress and by various stimuli. There are many reports indicating that ER stress plays an important role in the impairment of insulin biosynthesis, suggesting that reduction of ER stress could be a therapeutic target for diabetes. In this paper, we reviewed the relationship between ER stress and diabetes and how ER stress controls insulin biosynthesis.

## 1. Introduction

### 1.1. Diabetes and ER Stress

The endoplasmic reticulum (ER), a membrane compartment located near the nucleus, is the organelle where polypeptides, which will become secretory proteins or membrane proteins, are synthesized from mRNA and become mature proteins after undergoing folding, assembly, glycosylation, disulfide bonding, and posttranslational modifications [1]. The ER is well developed in endocrine cells such as pancreatic beta cells in which secretory proteins are synthesized. Proper functioning of the ER is essential to cell survival. ER stress is defined as an imbalance between client protein load and folding capacity and can be caused by multiple mechanisms including increases in improperly folded proteins, impairment of protein transport from the ER to the Golgi, inhibition of protein glycosylation, reduced disulfide bond formation, and calcium depletion of the ER lumen. When ER stress occurs, cellular defense mechanisms related to the ER stress response are activated. The ER stress response is comprised of (1) activation of the protein-kinase-RNA-(PKR-) like ER kinase (PERK) and reduction of protein translation by phosphorylation of the eukaryotic translation initiation factor 2 alpha (eIF2*α*); (2) activation of the inositol-requiring 1 (IRE1)/X-box- binding- protein- 1 (XBP-1) protein and the activating transcription factor 6 (ATF6) through the increased expression of ER chaperones and subsequent increase in ER folding capacity; (3) ER stress-associated protein degradation, which degrades unfolded or improperly folded proteins; (4) apoptosis by the activation of CCAAT/enhancer-binding homologous protein (CHOP) [2–4].

 Type 2 diabetes is characterized by insulin resistance and pancreatic beta cell dysfunction. Pancreatic beta cells compensate for insulin resistance by hypersecretion of insulin; however, at some point, pancreatic beta cells fail to secrete sufficient insulin, resulting in diabetes [5]. The pancreatic beta cells are susceptible to ER stress not only because of physiological variations in glucose levels that potently stimulate insulin translation but also because of other conditions that can cause ER stress such as metabolic dysregulation associated with obesity, including excess nutrients [6–10] and inflammatory cytokines [11–13]. Obesity can also contribute to insulin resistance via ER stress [14, 15]. In addition, many studies have shown that increased ER stress induces pancreatic beta cell dysfunction and diabetes mellitus using genetic models of ER stress [16–24].

### 1.2. Glucotoxicity or Lipotoxicity

For type 2 diabetic patients, a common clinical course is to respond to therapy initially by normalizing their fasting glucose levels, but then to undergo gradual deterioration in glycemic control, despite optimal medical management using a variety of drugs [25]. Although optimal management of type 2 diabetes regulates fasting glucose levels, most patients continue to have abnormally elevated postprandial glucose levels, which results in glucotoxicity [26]. Glucotoxicity is defined as nonphysiological and potentially irreversible pancreatic beta cell damage caused by chronic exposure to supraphysiological glucose concentrations [26]. Supraphysiological glucose concentrations also have adverse effects on cellular structure and function in tissues throughout the body. Chronic exposure to high glucose concentrations in HIT-T15 cells over 50 weeks caused a decrease in insulin gene transcription factors such as pancreatic-duodenal-homeobox-factor-1 (PDX-1) and MafA, insulin promoter activity, insulin mRNA, insulin content, and insulin secretion [27–31]. Glucotoxicity implies damage and irreversibility and should be differentiated from desensitization and exhaustion, which are reversible phenomena [32].

Obesity is a major predisposing factor for type 2 diabetes. The increase in free fatty acids (FFAs) due to obesity causes insulin resistance and, as a consequence, pancreatic beta cells secrete more insulin to compensate for insulin resistance and maintain normoglycemia. However, failure to compensate for insulin resistance by the pancreatic beta cells results in beta cell dysfunction and diabetes [5]. This phenomenon, which is a lipid-induced pancreatic beta cell dysfunction, is lipotoxicity. Chronic exposure to FFAs results in decreased insulin gene expression and proinsulin processing [33] and increased pancreatic beta cell death [34]. Recently, the concept of “glucolipotoxicity” has been introduced because lipotoxicity is dependent on elevated glucose levels and elevated glucose and FFAs have a synergistic effect on impairing pancreatic beta cell function [35]. Recently, increasing evidence has implicated ER stress in glucotoxicity and glucolipotoxicity in pancreatic beta cells [6, 9, 10].

### 1.3. Insulin Biosynthesis

Pancreatic-beta-cell-specific expression of the insulin gene requires both ubiquitous and cell-specific activators, which have target sequences within the enhancer located between −340 and −90 nucleotides relative to the transcription start site [36, 37]. Transcription of the insulin gene is regulated primarily by transcription factors such as PDX-1, BETA/NeuroD, and RIPE3b1/MafA. Under physiological conditions, MafA and PDX-1 bind to the C elements and the A boxes, respectively. Glucotoxicity greatly diminishes protein levels of PDX-1 through a posttranscriptional mechanism, and of MafA through a posttranslational mechanism. These abnormalities lead to decreases in insulin mRNA, insulin content, and glucose-induced insulin secretion [28, 30].

The ER plays an important role in the biosynthesis of insulin since the early steps of insulin biosynthesis occur in the ER [38]. The insulin gene encodes the preproinsulin polypeptide. Insulin is the posttranslational product of preproinsulin and is a globular protein containing two chains, A (21 residues) and B (30 residues). As preproinsulin is synthesized in the cytoplasm with a signal peptide, it is cotranslationally translocated into the lumen of the ER through the interaction between the signal peptide and the signal recognition particle on the ER membrane. The signal peptide of preproinsulin is cleaved in the ER and proinsulin is produced. In the lumen of the ER, proinsulin undergoes protein folding whereby three disulfide bonds are formed, which are essential for stability and bioactivity. Properly folded proinsulin is then delivered to the Golgi apparatus and packaged into secretory granules [39]. The conversion of proinsulin to insulin takes place in the secretory granules. Mature insulin is then released by exocytosis [40]. Therefore, ER stress due to increased misfolded proinsulin may induce beta cell dysfunction and diabetes.

Various conditions that are associated with diabetes mellitus, such as glucotoxicity and lipotoxicity, have been implicated in ER stress in pancreatic beta cells [41–43]. This paper focuses on the relationship between ER stress and insulin gene biosynthesis.

## 2. PERK/ATF4

When unfolded proteins accumulate in the ER lumen, the first response is to attenuate further translation of mRNAs, which reduces the ER load of new protein, preventing further accumulation of unfolded protein. This translational attenuation is mediated by PERK/eIF2*α* [19, 44]. In response to ER stress, eIF2*α* is phosphorylated by PERK. EIF2*α* is a heterotrimeric protein that is required to bring the initiator methionyl-transfer RNA (Met-tRNA) to the ribosome. PERK is a type I transmembrane serine/threonine kinase localized in the ER membrane. Under unstressed conditions, the ER chaperone Bip binds to the ER luminal domain of PERK and maintains this protein in an inactive form. Upon induction of ER stress, Bip binds to unfolded proteins and is thus competitively dissociated from PERK, leading to the activation of PERK by oligomerization and *trans*-autophosphorylation. Consequently, activated PERK phosphorylates eIF2*α* and inhibits translation [3, 44]. Phosphorylated eIF2*α* promotes expression of stress-induced genes, such as the transcription factors ATF4 and CHOP [22]. Moreover, in response to long-term adaptation to stress conditions, phosphorylation of eIF2*α* induces the expression of the growth arrest and DNA damage gene, GADD34. GADD34 is a stress-inducible regulatory subunit of a holophosphatase complex that dephosphorylates eIF2*α* together with protein phosphatase 1c (PP1c), and is an important component of translational recovery during the ER stress response [45, 46].

Many studies have reported the relationship between PERK and diabetes [18–20, 47–49]. Wolcott-Rallison syndrome indicates that the PERK gene is correlated with diabetes. Wolcott-Rallison syndrome is a rare human autosomal recessive genetic disorder characterized by early infancy type 1 diabetes resulting from mutations in the PERK gene [18, 47]. A similar phenotype has been described in PERK-/- mice [19]. The exocrine and endocrine pancreases develop normally in Perk-/- mice, but there is a progressive loss of insulin-producing pancreatic beta cells in the islets of Langerhans of Perk-/- mice postnatally, resulting in hyperglycemia and reduced serum insulin levels. Moreover, ER distention and activation of the ER stress transducer IRE1*α*, accompanied by increased cell death, leads to progressive diabetes mellitus and exocrine pancreatic insufficiency [19]. Zhang et al. reported that PERK-deficient mice exhibit severe defects in fetal/neonatal pancreatic beta cell proliferation and differentiation, resulting in low pancreatic beta cell mass, defects in proinsulin trafficking, and abrogation of insulin secretion, which together culminate in permanent neonatal diabetes [20]. Yusta B et al. reported that exendin-4, a GLP-1 receptor agonist, reduced the downregulation of insulin translation and improved cell survival under ER stress conditions. Exendin-4 increased ATF4 and CHOP expression and also potentiated the induction of GADD34 and PP1c activity, resulting in decreased phosphorylation of eIF2*α* and a faster recovery from translational repression. These findings show that GLP-1 receptor signaling modulates the ER stress response, leading to enhanced pancreatic beta cell survival [48].

## 3. IRE/XBP

The second response to ER stress is an increase in proteinfolding activity via the induction of ER chaperones such as Bip. This response is mediated by IRE1 and ATF6. IRE1 is a type I transmembrane endonuclease localized in the ER membrane. Similar to PERK, activation of IRE1 is triggered by dissociation of Bip from the IRE1 ER luminal domain, which leads to oligomerization and *trans*-autophosphorylation. Subsequently, IRE1 induces splicing of XBP-1, which upregulates unfolded protein response (UPR) target genes [2–4, 50]. IRE-XBP-1 signaling is important in secretory cells, such as exocrine pancreatic cells [51]. Lipson et al. reported that chronic hyperglycemia induces ER stress and activates IRE1, resulting in suppression of insulin expression at the transcriptional level [7]. Transient high glucose conditions induced the activation of IRE1*α* in pancreatic islet cells. Inactivation of IRE1*α* signaling by siRNA or inhibition of IRE1*α* phosphorylation decreases insulin biosynthesis under the transient high glucose conditions. However, IRE1 activation by high glucose concentrations was not accompanied by Bip dissociation and XBP-1 splicing, but IRE1 target genes were upregulated. These findings suggest that under transient high glucose conditions like postprandial hyperglycemia, IRE1*α* is activated and enhances proinsulin biosynthesis. By contrast, sustained activation of IRE1 signaling by chronic high glucose exposure causes ER stress, leading to the suppression of insulin mRNA expression. These findings suggested that sustained activation of IRE1*α* may decrease insulin biosynthesis at the transcriptional level. Overall, physiological IRE1*α* activation by transient high glucose conditions has a beneficial effect, but pathological IRE1*α* activation by chronic high glucose exposure is harmful to cells. In addition, under transient hyperglycemic conditions, activation of IRE1*α* was not accompanied by XBP-1 splicing, but long-term exposure to high glucose induced IRE1*α* activation and XBP-1 splicing, suggesting that XBP-1 splicing could be a marker of chronic hyperglycemic conditions [7]. Pirot et al. reported that IRE/XBP-1 increases degradation of insulin mRNA. Cyclopiazonic acid (CPA) is a sarcoendoplasmic reticulum Ca^2+^-ATPase (SERCA) blocker, which depletes ER calcium stores and induces ER stress. When INS-1 cells were treated with CPA, insulin mRNA levels decreased. Treatment with CPA did not affect insulin promoter activity, indicating that the decrease in insulin mRNA was not caused by a decrease in insulin transcription. By contrast, when cells were pretreated with actinomycin D, which arrests transcription, treatment with CPA induced a decrease in insulin expression that was due to mRNA degradation. Moreover, this study showed that insulin mRNA degradation in response to CPA was paralleled by an increase in IRE1 activation [52]. Another study has suggested that sustained production of spliced XBP-1 (XBP-1s) induces beta cell dysfunction by decreasing insulin gene expression, leading to apoptosis [53]. Adenoviral-mediated overproduction of XBP-1s resulted in increased XBP-1 activity and increased expression of XBP-1 target genes. XBP-1s overexpression impaired glucose-stimulated insulin secretion, increased beta cell apoptosis, and decreased levels of insulin, Pdx1, and Mafa mRNA. XBP-1s knockdown partially restored cytokine/ER-stress-driven insulin and Pdx1 inhibition. These data suggest that prolonged XBP-1s production induces beta cell dysfunction through inhibition of Pdx1, MafA, and insulin expression leading to beta cell apoptosis [53].

## 4. ATF6

ATF 6 is another mediator of ER- stress-induced transcription. ATF6 is a member of the ATF, cAMP response element-binding protein, basic-leucine zipper (bZIP) DNA-binding protein family of transcriptional activators [54]. ATF6 is a 90 kDa protein (p90ATF6) of 670 amino acids [55] and contains a transmembrane domain (amino acids 378–398) with the N terminus facing the cytoplasm [56]. In the unstressed state, p90ATF6 is localized to the ER [56]. In response to ER stress, ATF6 translocates from the ER to the Golgi [56, 57], where it is cleaved by site 1 and site 2 proteases [58]. Proteolytic cleavage of ATF6 removes the N-terminal cytosolic domain, which is transported into the nucleus and directly induces transcriptional activation of chaperone molecules such as BIP/GRP78 and other enzymes that are essential for protein folding [56, 58–60]. Moreover, activated ATF6 directly binds to the ER stress response element (ERSE) and induces XBP-1 expression.

The spliced form of XBP-1 is produced from the upregulated XBP-1 mRNA by activation of IRE1*α* and binds to ERSE sequences directly. When ER stress occurs, activation of ATF6 is rapid because it is produced from a preexisting precursor protein, whereas activation of XBP-1 is slow because XBP-1 mRNA must be induced, spliced, and translated to produce an active form of XBP-1. This observation suggests that ATF6 is activated early in response to ER stress and XBP-1 functions in more sustained ER stress [61, 62]. A prolonged increase in the demand for insulin often leads to defects in insulin secretion, resulting in sustained hyperglycemia [63]. Chronic hyperglycemia has deleterious effects on beta cell function, as shown in primary cultured rat and human islet cells and in beta cell lines [64].

Previously, our studies confirmed that chronic high glucose induces beta cell dysfunction. Under basal glucose concentrations (5 mmol/l glucose), insulin mRNA is expressed abundantly in INS-1 cells, but, in the presence of 30 mmol/l glucose, insulin mRNA expression was decreased in a time-dependent manner until only minimal levels were detected by 96 h. The expression of PDX-1 and MafA, which are the main transcription factors associated with insulin gene expression, also decreased over time in the 30 mmol/l glucose [65]. In addition, chronic hyperglycemia induced ER- stress in cultured INS-1 cells and ER stress-induced activation of ATF6 impaired insulin gene expression via upregulation of the orphan nuclear receptor small heterodimer partner (SHP). Prolonged exposure of INS-1 cells to a high concentration of glucose increased ER stress in the cells, and ER stress induced by the chemical ER stressors tunicamycin, thapsigargin, and DTT impaired insulin gene expression. Among the three different signaling pathways of the ER stress response (ATF6, IRE1, and PERK), ATF6 inhibited insulin promoter activity, but IRE1-XBP1 and PERK-eIF2*α*-ATF4 did not. Adenovirus-mediated overexpression of the active form of ATF6 (Ad-ATF6) in INS-1 cells down-regulated PDX-1 and MafA gene expression and repressed the cooperative action of PDX-1, BETA2, and MafA in stimulating insulin transcription [66].

Several *in vitro* studies have shown that SHP negatively regulates insulin biosynthesis and secretion in pancreatic beta cells [67]. SHP also directly represses the transcriptional activity of the basic helix-loop-helix transcription factor BETA2, a positive regulator of insulin gene expression [68], and indirectly represses p300-enhanced BETA2/NeuroD transcriptional activity through inhibition of the BETA2-p300 interaction [69]. Previously, we reported that glucotoxicity in INS-1 cells is mediated by SHP. Culture of INS-1 cells and rat pancreatic islets in the continuous presence of high glucose concentrations increased SHP mRNA expression, followed by a decrease in insulin gene expression. Furthermore, adenovirus-mediated overexpression of SHP in INS-1 cells impaired glucose-stimulated insulin secretion as well as insulin gene expression [65]. Interestingly, Ad-ATF6 increased SHP gene expression, and downregulation of endogenous SHP expression by siRNA-SHP blocked ATF6-induced suppression of insulin gene expression. These data suggest that ER-stress-induced beta cell dysfunction is mediated, at least in part, by ATF6-induced transcriptional activation of SHP. Collectively, chronic high glucose exposure induced ER stress, and ER stress-induced activation of ATF6 resulted in beta cell dysfunction mediated, in part, by the upregulation of SHP expression [66].

## 5. Conclusions

**Figure 1:**
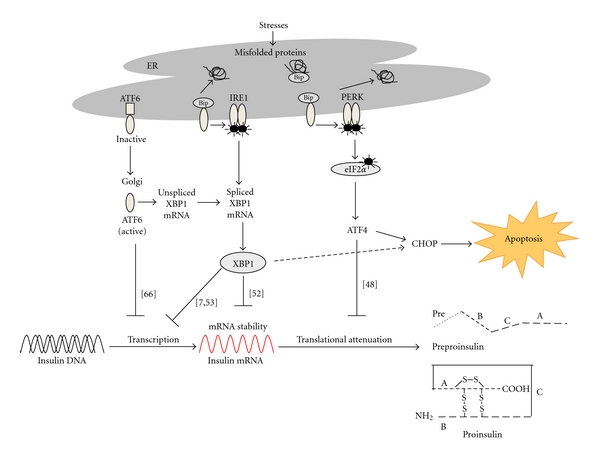
Possible mechanisms of insulin biosynthesis by endoplasmic reticulum (ER) stress. Insulin biosynthesis is affected by ER stress through different mechanisms, such as activation of the following pathways: transcription factor 6 (ATF6), inositol-requiring 1 (IRE1)/X-box-binding-protein-1 (XBP-1), and protein-kinase-RNA (PKR-) like ER kinase (PERK). The exposure of beta cells to high glucose chronically induces ER stress, resulting in the activation of ATF6. Activation of ATF6 impairs insulin gene expression. Long-term exposure to high glucose induces IRE1*α* activation and XBP-1 splicing, leading to the suppression of insulin mRNA expression and to increases in the degradation of insulin mRNA. In addition, downstream of PERK, ATF4, and CHOP inhibit proinsulin synthesis via translational attenuation mediated by PP1c and GADD34. Numbers in parentheses are the references cited in this paper.

Many studies have reported that ER stress plays an important role in the pathogenesis of diabetes and that the UPR has an important role in regulating pancreatic beta cell functions. These studies have suggested that resolving ER stress could be a therapeutic target for diabetes. Moreover, it has been suggested from experimental evidence that ER stress mediates glucolipotoxicity-induced suppression of insulin biosynthesis (Figure 1). In addition to impaired insulin biosynthesis, understanding impaired insulin secretion and beta cell failure, including apoptosis in glucolipotoxicity-induced ER stress conditions, could have important implications for the development of therapeutic strategies for type 2 diabetes mellitus. However, we do not know completely how ER stress affects the pathogenesis of human diabetes. To clarify the role of ER stress in insulin biosynthesis, investigations to determine whether ER stress is implicated in the development of human diabetes and the interaction of the three arms of ER stress are needed. Besides, it will be important for future studies to address the relationship between the ER stress response gene and human disease by performing a genetic study.

## References

[1] Kaufman RJ (1999). Stress signaling from the lumen of the endoplasmic reticulum: coordination of gene transcriptional and translational controls. *Genes and Development*.

[2] Mori K (2000). Tripartite management of unfolded proteins in the endoplasmic reticulum. *Cell*.

[3] Kaufman RJ, Scheuner D, Schröder M (2002). The unfolded protein response in nutrient sensing and differentiation. *Nature Reviews Molecular Cell Biology*.

[4] Oyadomari S, Araki E, Mori M (2002). Endoplasmic reticulum stress-mediated apoptosis in pancreatic *β*-cells. *Apoptosis*.

[5] Kasuga M (2006). Insulin resistance and pancreatic *β* cell failure. *Journal of Clinical Investigation*.

[6] Wang H, Kouri G, Wollheim CB (2005). ER stress and SREBP-1 activation are implicated in B-cell glucolipotoxicity. *Journal of Cell Science*.

[7] Lipson KL, Fonseca SG, Ishigaki S (2006). Regulation of insulin biosynthesis in pancreatic beta cells by an endoplasmic reticulum-resident protein kinase IRE1. *Cell Metabolism*.

[8] Lipson KL, Ghosh R, Urano F (2008). The role of IRE1*α* in the degradation of insulin mRNA in pancreatic *β*-cells. *PLoS ONE*.

[9] Kharroubi I, Ladrière L, Cardozo AK, Dogusan Z, Cnop M, Eizirik DL (2004). Free fatty acids and cytokines induce pancreatic *β*-cell apoptosis by different mechanisms: role of nuclear factor-*κ*B and endoplasmic reticulum stress. *Endocrinology*.

[10] Laybutt DR, Preston AM, Åkerfeldt MC (2007). Endoplasmic reticulum stress contributes to beta cell apoptosis in type 2 diabetes. *Diabetologia*.

[11] Oyadomari S, Takeda K, Takiguchi M (2001). Nitric oxide-induced apoptosis in pancreatic *β* cells is mediated by the endoplasmic reticulum stress pathway. *Proceedings of the National Academy of Sciences of the United States of America*.

[12] Cardozo AK, Ortis F, Storling J (2005). Cytokines downregulate the sarcoendoplasmic reticulum pump Ca2+ ATPase 2b and deplete endoplasmic reticulum Ca2+, leading to induction of endoplasmic reticulum stress in pancreatic *β*-cells. *Diabetes*.

[13] Chan JY, Cooney GJ, Biden TJ, Laybutt DR (2011). Differential regulation of adaptive and apoptotic unfolded protein response signalling by cytokine-induced nitric oxide production in mouse pancreatic beta cells. *Diabetologia*.

[14] Özcan U, Cao Q, Yilmaz E (2004). Endoplasmic reticulum stress links obesity, insulin action, and type 2 diabetes. *Science*.

[15] Hotamisligil GS (2005). Role of endoplasmic reticulum stress and c-Jun NH2-terminal kinase pathways in inflammation and origin of obesity and diabetes. *Diabetes*.

[16] Yoshioka M, Kayo T, Ikeda T, Koizumi A (1997). A novel locus, Mody4, distal to D7Mit189 on chromosome 7 determines early-onset NIDDM in nonobese C57BL/6 (Akita) mutant mice. *Diabetes*.

[17] Wang J, Takeuchi T, Tanaka S (1999). A mutation in the insulin 2 gene induces diabetes with severe pancreatic *β*-cell dysfunction in the Mody mouse. *Journal of Clinical Investigation*.

[18] Delépine M, Nicolino M, Barrett T, Golamaully M, Mark Lathrop G, Julier C (2000). EIF2AK3, encoding translation initiation factor 2-*α* kinase 3, is mutated in patients with Wolcott-Rallison syndrome. *Nature Genetics*.

[19] Harding HP, Zeng H, Zhang Y (2001). Diabetes mellitus and exocrine pancreatic dysfunction in Perk-/- mice reveals a role for translational control in secretory cell survival. *Molecular Cell*.

[20] Zhang W, Feng D, Li Y, Iida K, McGrath B, Cavener DR (2006). PERK EIF2AK3 control of pancreatic *β* cell differentiation and proliferation is required for postnatal glucose homeostasis. *Cell Metabolism*.

[21] Scheuner D, Mierde DV, Song B (2005). Control of mRNA translation preserves endoplasmic reticulum function in beta cells and maintains glucose homeostasis. *Nature Medicine*.

[22] Scheuner D, Song B, McEwen E (2001). Translational control is required for the unfolded protein response and in vivo glucose homeostasis. *Molecular Cell*.

[23] Meur G, Simon A, Harun N (2010). Insulin gene mutations resulting in early-onset diabetes: marked differences in clinical presentation, metabolic status, and pathogenic effect through endoplasmic reticulum retention. *Diabetes*.

[24] Fonseca SG, Ishigaki S, Oslowski CM (2010). Wolfram syndrome 1 gene negatively regulates ER stress signaling in rodent and human cells. *Journal of Clinical Investigation*.

[25] UK Prospective Diabetes Study (UKPDS) Group (1998). Effect of intensive blood-glucose control with metformin on complications in overweight patients with type 2 diabetes (UKPDS 34). *The Lancet*.

[26] Robertson RP, Harmon J, Tran POT, Poitout V (2004). *β*-cell glucose toxicity, lipotoxicity, and chronic oxidative stress in type 2 diabetes. *Diabetes*.

[27] Robertson RP, Zhang HJ, Pyzdrowski KL, Walseth TF (1992). Preservation of insulin mRNA levels and insulin secretion in HIT cells by avoidance of chronic exposure to high glucose concentrations. *Journal of Clinical Investigation*.

[28] Olson LK, Redmon JB, Towle HC, Robertson RP (1993). Chronic exposure of HIT cells to high glucose concentrations paradoxically decreases insulin gene transcription and alters binding of insulin gene regulatory protein. *Journal of Clinical Investigation*.

[29] Moran A, Zhang HJ, Olson LK, Harmon JS, Poitout V, Robertson RP (1997). Differentiation of glucose toxicity from beta cell exhaustion during the evolution of defective insulin gene expression in the pancreatic islet cell line, HIT-T15. *Journal of Clinical Investigation*.

[30] Olson LK, Sharma A, Peshavaria M (1995). Reduction of insulin gene transcription in HIT-T15 *β* cells chronically exposed to a supraphysiologic glucose concentration is associated with loss of STF-1 transcription factor expression. *Proceedings of the National Academy of Sciences of the United States of America*.

[31] Sharma A, Olson LK, Robertson RP, Stein R (1995). The reduction of insulin gene transcription in HIT-T15 *β* cells chronically exposed to high glucose concentration is associated with the loss of RIPE3b1 and STF-1 transcription factor expression. *Molecular Endocrinology*.

[32] Robertson RP, Olson LK, Zhang HJ (1994). Differentiating glucose toxicity from glucose desensitization: a new message from the insulin gene. *Diabetes*.

[33] Bollheimer LC, Skelly RH, Chester MW, McGarry JD, Rhodes CJ (1998). Chronic exposure to free fatty acid reduces pancreatic *β* cell insulin content by increasing basal insulin secretion that is not compensated for by a corresponding increase in proinsulin biosynthesis translation. *Journal of Clinical Investigation*.

[34] Shimabukuro M, Higa M, Zhou YT, Wang MY, Newgard CB, Unger RH (1998). Lipoapoptosis in beta-cells of obese prediabetic fa/fa rats. Role of serine palmitoyltransferase overexpression. *Journal of Biological Chemistry*.

[35] Prentki M, Joly E, El-Assaad W, Roduit R (2002). Malonyl-CoA signaling, lipid partitioning, and glucolipotoxicity: role in *β*-cell adaptation and failure in the etiology of diabetes. *Diabetes*.

[36] Qiu Y, Guo M, Huang S, Stein R (2002). Insulin gene transcription is mediated by interactions between the p300 coactivator and PDX-1, BETA2, and E47. *Molecular and Cellular Biology*.

[37] Sander M, German MS (1997). The *β* cell transcription factors and development of the pancreas. *Journal of Molecular Medicine*.

[38] Harding HP, Ron D (2002). Endoplasmic reticulum stress and the development of diabetes: a review. *Diabetes*.

[39] Xue Fen Huang, Arvan P (1994). Formation of the insulin-containing secretory granule core occurs within immature *β*-granules. *Journal of Biological Chemistry*.

[40] Weiss MA (2009). Proinsulin and the genetics of diabetes mellitus. *Journal of Biological Chemistry*.

[41] Eizirik DL, Cardozo AK, Cnop M (2008). The role for endoplasmic reticulum stress in diabetes mellitus. *Endocrine Reviews*.

[42] Jang YY, Kim NK, Kim MK (2010). The effect of tribbles-related protein 3 on ER stress-suppressed insulin gene expression in INS-1 cells. *Korean Diabetes Journal*.

[43] Scheuner D, Kaufman RJ (2008). The unfolded protein response: a pathway that links insulin demand with *β*-cell failure and diabetes. *Endocrine Reviews*.

[44] Harding HP, Zhang Y, Ron D (1999). Protein translation and folding are coupled by an endoplasmic- reticulum-resident kinase. *Nature*.

[45] Novoa I, Zhang Y, Zeng H, Jungreis R, Harding HP, Ron D (2003). Stress-induced gene expression requires programmed recovery from translational repression. *EMBO Journal*.

[46] Novoa I, Zeng H, Harding HP, Ron D (2001). Feedback inhibition of the unfolded protein response by GADD34-mediated dephosphorylation of eIF2*α*. *Journal of Cell Biology*.

[47] Wolcott CD, Rallison ML (1972). Infancy-onset diabetes mellitus and multiple epiphyseal dysplasia. *The Journal of Pediatrics*.

[48] Yusta B, Baggio LL, Estall JL (2006). GLP-1 receptor activation improves *β* cell function and survival following induction of endoplasmic reticulum stress. *Cell Metabolism*.

[49] Back SH, Scheuner D, Han J (2009). Translation Attenuation through eIF2*α* Phosphorylation Prevents Oxidative Stress and Maintains the Differentiated State in *β* Cells. *Cell Metabolism*.

[50] Bertolotti A, Zhang Y, Hendershot LM, Harding HP, Ron D (2000). Dynamic interaction of BiP and ER stress transducers in the unfolded-protein response. *Nature Cell Biology*.

[51] Calfon M, Zeng H, Urano F (2002). IRE1 couples endoplasmic reticulum load to secretory capacity by processing the XBP-1 mRNA. *Nature*.

[52] Pirot P, Naamane N, Libert F (2007). Global profiling of genes modified by endoplasmic reticulum stress in pancreatic beta cells reveals the early degradation of insulin mRNAs. *Diabetologia*.

[53] Allagnat F, Christulia F, Ortis F (2010). Sustained production of spliced X-box binding protein 1 (XBP1) induces pancreatic beta cell dysfunction and apoptosis. *Diabetologia*.

[54] Hai T, Liu F, Coukos WJ, Green MR (1989). Transcription factor ATF cDNA clones: an extensive family of leucine zipper proteins able to selectively form DNA-binding heterodimers. *Genes and Development*.

[55] Zhu C, Johansen FE, Prywes R (1997). Interaction of ATF6 and serum response factor. *Molecular and Cellular Biology*.

[56] Haze K, Yoshida H, Yanagi H, Yura T, Mori K (1999). Mammalian transcription factor ATF6 is synthesized as a transmembrane protein and activated by proteolysis in response to endoplasmic reticulum stress. *Molecular Biology of the Cell*.

[57] Chen X, Shen J, Prywes R (2002). The luminal domain of ATF6 senses endoplasmic reticulum (ER) stress and causes translocation of ATF6 from the er to the Golgi. *Journal of Biological Chemistry*.

[58] Ye J, Rawson RB, Komuro R (2000). ER stress induces cleavage of membrane-bound ATF6 by the same proteases that process SREBPs. *Molecular Cell*.

[59] Yoshida H, Okada T, Haze K (2000). ATF6 activated by proteolysis binds in the presence of NF-Y (CBF) directly to the cis-acting element responsible for the mammalian unfolded protein response. *Molecular and Cellular Biology*.

[60] Wang Y, Shen J, Arenzana N, Tirasophon W, Kaufman RJ, Prywes R (2000). Activation of ATF6 and an ATF6 DNA binding site by the endoplasmic reticulum stress response. *Journal of Biological Chemistry*.

[61] Yoshida H, Matsui T, Yamamoto A, Okada T, Mori K (2001). XBP1 mRNA is induced by ATF6 and spliced by IRE1 in response to ER stress to produce a highly active transcription factor. *Cell*.

[62] Yoshida H, Matsui T, Hosokawa N, Kaufman RJ, Nagata K, Mori K (2003). A time-dependent phase shift in the mammalian unfolded protein response. *Developmental Cell*.

[63] Koh EH, Kim MS, Park JY (2003). Peroxisome proliferator-activated receptor (PPAR)-*α* activation prevents diabetes in OLETF rats: comparison with PPAR-*γ* activation. *Diabetes*.

[64] Ross Laybutt D, Sharma A, Sgroi DC, Gaudet J, Bonner-Weir S, Weir GC (2002). Genetic regulation of metabolic pathways in *β*-cells disrupted by hyperglycemia. *Journal of Biological Chemistry*.

[65] Park KG, Lee KM, Seo HY (2007). Glucotoxicity in the INS-1 rat insulinoma cell line is mediated by the orphan nuclear receptor small heterodimer partner. *Diabetes*.

[66] Seo HY, Yong DK, Lee KM (2008). Endoplasmic reticulum stress-induced activation of activating transcription factor 6 decreases insulin gene expression via up-regulation of orphan nuclear receptor small heterodimer partner. *Endocrinology*.

[67] Yamagata K, Furuta H, Oda N (1996). Mutations in the hepatocyte nuclear factor-4*α* gene in maturity-onset diabetes of the young (MODY1). *Nature*.

[68] Naya FJ, Stellrecht CMM, Tsai MJ (1995). Tissue-specific regulation of the insulin gene by a novel basic helix- loop-helix transcription factor. *Genes and Development*.

[69] Kim JY, Chu K, Kim HJ (2004). Orphan nuclear receptor small heterodimer partner, a novel corepressor for a basic helix-loop-helix transcription factor BETA2/NeuroD. *Molecular Endocrinology*.

